# The potential of polymeric micelles in the context of glioblastoma therapy

**DOI:** 10.3389/fphar.2013.00157

**Published:** 2013-12-30

**Authors:** Ramin A. Morshed, Yu Cheng, Brenda Auffinger, Michelle L. Wegscheid, Maciej S. Lesniak

**Affiliations:** The Brain Tumor Center, The University of Chicago Pritzker School of MedicineChicago, IL, USA

**Keywords:** glioblastoma, micelles, nanoparticle, drug delivery, targeted delivery, controlled release

## Abstract

Glioblastoma multiforme (GBM), a type of malignant glioma, is the most common form of brain cancer found in adults. The current standard of care for GBM involves adjuvant temozolomide-based chemotherapy in conjunction with radiotherapy, yet patients still suffer from poor outcomes with a median survival of 14.6 months. Many novel therapeutic agents that are toxic to GBM cells *in vitro* cannot sufficiently accumulate at the site of an intracranial tumor after systemic administration. Thus, new delivery strategies must be developed to allow for adequate intratumoral accumulation of such therapeutic agents. Polymeric micelles offer the potential to improve delivery to brain tumors as they have demonstrated the capacity to be effective carriers of chemotherapy drugs, genes, and proteins in various preclinical GBM studies. In addition to this, targeting moieties and trigger-dependent release mechanisms incorporated into the design of these particles can promote more specific delivery of a therapeutic agent to a tumor site. However, despite these advantages, there are currently no micelle formulations targeting brain cancer in clinical trials. Here, we highlight key aspects of the design of polymeric micelles as therapeutic delivery systems with a review of their clinical applications in several non-brain tumor cancer types. We also discuss their potential to serve as nanocarriers targeting GBM, the major barriers preventing their clinical implementation in this disease context, as well as current approaches to overcome these limitations.

## INTRODUCTION

Malignant gliomas are the most common primary brain tumors found in adult patients and are comprised of astrocytomas, oligodendrogliomas, and ependymomas (Maher et al., 2001). Of these tumor types, the most frequent and deadly is glioblastoma multiforme (GBM), a grade IV astrocytoma. As demonstrated in Stupp et al. (2005), patients with GBM exhibit a median survival of 14.6 months and a 5 years survival rate of less than 10% (Stupp et al., 2009) after undergoing radiotherapy with adjuvant temozolomide. Not much has changed in the standard-of-care for these patients over the last decade. Nanoparticle agents such a liposomal doxorubicin, anti-angiogenic agents such as bevacizumab, oncolytic viruses, and several other agents have been employed with modest clinical benefit (Hau et al., 2004; Friedman et al., 2009; Westphal et al., 2013; Zustovich et al., 2013). Novel delivery methods such as convection-enhanced delivery (CED) have also been applied to patients to improve distribution of an intratumorally administered drug (Kunwar et al., 2010; White et al., 2012). Yet, a “cure” for this disease is still non-existent, demonstrating the need to improve our understanding of the underlying disease process in GBM as well as the need to explore new avenues of therapeutic delivery.

Nanomedicines demonstrate great promise for the delivery of chemotherapy agents and other small biomolecules. These minute particles possess a variety of functions depending on the system in question, offering longer drug circulation times, greater delivery specificity, and greater penetration into solid tumors (Tiwari and Amiji, 2006; Fang et al., 2011). More and more nanoparticle systems are being brought to cancer patients in a clinical setting. For example, liposomal formulations of doxorubicin (e.g., Doxil^®^, Caelyx^®^) are now currently used for patients with a variety of cancers (Northfelt et al., 1998; Gordon et al., 2004; O’Brien et al., 2004; Orlowski et al., 2007) and are even now being tested for efficacy in patients with GBM and brain metastases from solid tumors (Caraglia et al., 2006; Beier et al., 2009). Iron oxide particles too are starting to be used for thermotherapy and imaging purposes in GBM patients (Enochs et al., 1999; Maier-Hauff et al., 2010)

Polymeric micelles are another group of nanoparticles that are making their way into the clinical arena (Gong et al., 2012). These amphiphilic nanoparticles have demonstrated the ability to deliver several different types of therapeutic agents, including chemotherapy drugs, proteins, siRNA, and DNA to tumor cells (Yoo et al., 2002; Oba et al., 2007; Heffernan and Murthy, 2009; Tong et al., 2010; Zhan et al., 2010, 2012a,b; Gao et al., 2012; Yin et al., 2013; Zheng et al., 2013). Not only are micelles highly biocompatible, they are also very flexible in terms of design modification. This can allow for the incorporation of a range of drug release mechanisms and targeting moieties into their structure. There are a number of micelle formulations that are currently being used to target several different cancer types in a clinical setting. However, to date, none of these formulations tested aim to treat patients with GBM or any other brain tumors for that matter. As these platforms have shown very promising results for a list of other solid tumors, it is important to examine what the current barriers are to transforming these particles into delivery systems that may one day benefit GBM patients.

In this review, we first examine the composition and characteristics of polymeric micelles as well as common methods of drug incorporation. We next detail micelle formulations used in clinical trials involving non-brain tumor cancers as well as the major limitations preventing their application as therapeutics for GBM. Finally, we discuss recent progress in the field of micellar design as well as methods for nanoparticle delivery that may help overcome some of these limitations and potentially allow for the targeting of high-grade malignant glioma.

## DESIGN OF POLYMERIC MICELLES

### COMPOSITION AND CHARACTERISTICS

Polymeric micelles are prepared from spontaneously self-assembling amphiphilic block copolymers in aqueous medium. In general, these block copolymers consist of either two (hydrophilic-hydrophobic) or three (hydrophilic-hydrophobic-hydrophilic) segments. Assembly of the hydrophobic components of these copolymers creates a hydrophobic core that is separated from the aqueous environment by hydrophilic segments (Chen and Jiang, 2005). Hydrophobic interactions act as the principle driving force for micelle formation, but other intermolecular forces including hydrogen bonding, electrostatic interaction, and metal complexation have also been applied to increase stability (Harada and Kataoka, 1995; Kabanov et al., 1996, Kataoka et al., 1998; Nishiyama et al., 2003). Ionic copolymers may also be used, allowing for the formation of electrostatically stabilized polyion complex micelles (Harada and Kataoka, 1998, 2003; Kataoka et al., 1999) and polymer-metal complex micelles (Nishiyama et al., 2001, 2003).

Polyesters, poly(amino acids), and polyethers are commonly used as hydrophobic or ionic segments (Matsumura, 2008; Bae and Kataoka, 2009; Osada et al., 2009; Zhang et al., 2010; Gao et al., 2012; Tao et al., 2012). An advantage of polyesters, including poly(D,L-lactide) (PLA), and poly(glycolic acid) (PGA), and poly(ε-caprolactone) (PCL), is that they are FDA approved for biomedical applications owing to their biocompatibility and biodegradability (Gaucher et al., 2005). Poly(amino acids) such as poly(α,β-aspartic acid) (PAsp) and poly(L-lysine) (PLys) have also been extensively used to form the core of micelles via hydrophobic and electrostatic interactions (Lavasanifar et al., 2002; Matsumura et al., 2004). The variety of functional groups on poly(amino acids) facilitate numerous possibilities for drug-conjugation (Kataoka et al., 2001) as well as DNA incorporation into the core of micelles (Katayose and Kataoka, 1997).

The outer coating polymers are also essential components to stabilize the micellar structure in aqueous solution. Of these, polyethylene glycol (PEG) is most commonly used for the shell formation (Bae and Kataoka, 2009; Osada et al., 2009; Zhan et al., 2010; Shen et al., 2011; Gao et al., 2012). PEG is a non-toxic polymer with low immunogenicity that been widely used for prolonging the circulation time of drug-delivery systems (Verrecchia et al., 1995; Huang et al., 2012). It can prevent protein adsorption and minimize non-specific uptake of nanoparticles by the reticuloendothelial system in the body (Huang et al., 2012). Alternative biocompatible hydrophilic polymers to PEG include dextran, chitosan, and poly(ethylenimide) (PEI), which have also been used in the formation of the hydrophilic corona (Kwon and Kataoka, 1995; Qiu and Bae, 2007; Verma et al., 2012; Xie et al., 2012).

Other characteristics of polymeric micelles must also be considered when designing them for appropriate clinical use. Compared to surfactant micelles, polymeric micelles possess a lower critical micelle concentration (CMC), greater biocompatibility, and improved stability (Miller et al., 2012). The CMC of these systems is especially important to keep in mind. Below the CMC value, micelles begin to dissociate into monomers, decreasing the longevity of these particles *in vivo*. Especially in the context of targeting a brain tumor, these particles must remain intact for a sufficient amount of time in order to penetrate into and fully distribute within the tumor site. The CMC of a micelle depends to a significant extent on the copolymers used as well as conjugates incorporated within its structure, with values ranging from 10^-^^6^ to 10^-^^7^ M (Bae and Kataoka, 2009; Huang et al., 2012).

It is also critical to consider size and charge when designing micellar systems because the blood brain barrier (BBB) can regulate entry into the central nervous system (CNS) based on these characteristics. The BBB is comprised of tight junctions between endothelial cells, a number of transporters including efflux proteins (e.g. P-glycoprotein), as well as surrounding astrocytes that modulate endothelial function (Abbott and Romero, 1996). It acts to exclude many toxic and infectious agents, yet it remains a major barrier in the passage of chemotherapy agents into the CNS. The size of polymeric micelles typically ranges from about 10–100 nm depending on the composition and synthesis method (Huang et al., 2012). Particles even larger than this have been shown to penetrate the disrupted BBB and accumulate within tumor tissue by the enhanced permeability and retention (EPR) effect (Iyer et al., 2007). This is accomplished by diffusion of micelles out of circulation due to a tumor’s disrupted vasculature and accumulation within the tumor area due to poor drainage of interstitial fluid (Maeda, 2001). In terms of body clearance of these particles, micelles are large enough to prevent rapid renal clearance, which normally limits the effectiveness of smaller drug molecules less than 40 kDa (Greish et al., 2003).

### METHODS OF THERAPEUTIC AGENT INCORPORATION

Typically, the hydrophobic core of micelles serves as the site of therapeutic agent incorporation. As stated previously, these therapeutic agents include chemotherapy drugs, proteins, genes, and siRNA (Yoo et al., 2002; Oba et al., 2007; Heffernan and Murthy, 2009; Tong et al., 2010; Zhan et al., 2010, 2012a,b; Gao et al., 2012; Yin et al., 2013; Zheng et al., 2013) but can even include other nanoparticles such as superparamagnetic iron oxide (SPIO) particles (Nasongkla et al., 2006; Kessinger et al., 2011). The method of incorporation is dependent on the desired function and agent in question. For example, doxorubicin is incorporated non-covalently into the core of micelle in the SP1049C formulation used in clinical trials (Danson et al., 2004) whereas incorporation of cisplatin into NC-6004, another micelle formulation used in clinical trials, relies on the substation of Pt(II) atom from chloride to carboxylate in the side chain of poly(Glu). Nasongkla et al. (2006) was able to incorporate doxorubicin and SPIO nanoparticles into the core of micelles, both via non-covalent interactions. Genes can be incorporated into the core of micelles via electrostatic interaction with positively charge copolymer components such as PEI (Pathak et al., 2009; Zhan et al., 2012b). Micelles have even been coated with gold nanoparticles via shell-crosslinking, which could allow for the attachment of therapeutic or targeting agents to this surface of the particle in addition to loading within their core (Bae et al., 2006). Stimulus-sensitive linker molecules can also play a role in therapeutic agent incorporation but will be discussed later on in this review.

## MICELLAR FORMULATIONS IN CLINICAL TRIALS

A number of clinical trials over the last decade have used polymeric micelles as carriers of potent chemotherapy agents to target many types of solid tumors. In the following section, we summarize the results of these clinical studies with an emphasis on the efficacy of various micellar formulations.

### GENEXOL-PM

Genexol-PM is a micellar formulation composed of monomethoxy-PEG-block-poly(D,L-lactide) copolymer with paclitaxel loaded into its hydrophobic core (Gong et al., 2012). A major advantage of this formulation is that it does not contain Cremophor EL, a toxic surfactant normally used to solubilize paclitaxel in circulation in the clinical formulation of Taxol. There have been a number of Phase I and II clinical studies examining the use of Genexol-PM in solid tumor treatment. In a phase I clinical study, Kim et al. (2004) used Genexol-PM to treat patients with lung, colorectal, renal cell, breast, ovarian, and esophageal cancers. Out of 21 patients, 3 (14.3%) achieved partial responses and 6 (28.6%) maintained stable disease. Dose-limiting toxicities included neutropenia, sensory neuropathy, and myalgia (Kim et al., 2004). Lim et al. (2010) utilized a different dosing regimen (weekly delivery instead of once every 3 weeks) of Genexol-PM to treat patients with breast, head and neck, lung, and nasopharyngeal cancer. Out of 21 patients, 5 (23.8%) achieved partial responses and 9 (42.9%) maintained stable disease (Lim et al., 2010). A phase II clinical trial examining the efficacy of Genexol-PM in patients with metastatic breast cancer demonstrated that out of 41 patients, 5 (12.2%) achieved complete responses, 19 (46.3%) achieved partial responses, and 13 (31.7%) maintained stable disease (Lee et al., 2008). The median overall survival in this study was not reached, even with a median follow-up time of 17 months. It would have been interesting to see this formulation’s effect on brain metastasis in this disease context; however, one of the exclusion criteria in the study was CNS metastases (Lee et al., 2008). In another phase II clinical study, Kim et al. (2007) used a combination of Genexol-PM and cisplatin to treat advanced non-small cell lung cancer. Out of 69 patients, 26 (37.7%) achieved a partial response and 20 (29.0%) maintained stable disease. The study reported a median overall survival period of 21.7 months. Because this formulation lacks Cremophor EL, the authors pointed out that higher doses of paclitaxel could be achieved without an increase in toxicity (Kim et al., 2007). Genexol-PM was also tested in a phase II clinical trial against advanced pancreatic cancer (Saif et al., 2010). Median overall survival was 6.5 months for patients treated with a dose of 300 or 350 mg/m^2^. For the 45 patients treated with this dose, 1 (2.2%) achieved a complete response, 2 (4.4%) achieved a partial response, and 24 (53.3%) maintained stable disease.

### NK105

Genexol-PM is not the only clinical micelle formulation containing paclitaxel. NK105 is a core-shell micelle composed of PEG and poly(aspartic acid) modified with 4-phenyl-1-butanol to increase its hydrophobicity (Gong et al., 2012). In a preclinical study, Hamaguchi et al. (2005) investigated treatment efficacy in a colorectal cancer xenograft mouse model. NK105 exerted superior anti-tumor activity as compared to free paclitaxel in nude mice transplanted with HT-29 colon cancer cells, and a 25-fold higher tumor area under the curve (AUC) for NK105 was observed compared to free paclitaxel (Hamaguchi et al., 2005). In a phase I clinical study, Hamaguchi et al. (2007) looked at treatment with NK105 in pancreatic, bile duct, and colon cancer. Out of 19 patients, 6 (31.6%) patients were found to have stable disease, and a partial response was seen in 1 patient with metastatic pancreatic cancer and 1 patient with metastatic stomach cancer (10.5% in total). AUC and total clearance rate of NK105 at 150 mg/m^2^ were ~32-fold larger and 72-fold lower, respectively, than for Genexol-PM at a dose of 300 mg/m^2^, suggesting NK105 is more stable in circulation (Hamaguchi et al., 2007). NK105 was also used to treat patients with advanced or recurrent gastric cancer in a phase II clinical trial (Kato et al., 2012). Out of 56 patients evaluable for efficacy, 2 (3.6%) achieved complete responses, 12 (21.4%) achieved partial responses, and 17 (30.4%) maintained stable disease. Median overall survival was 14.4 months. A phase III clinical trial using NK105 to combat breast cancer was begun in July 2012, but no updates have been presented thus far (NCT01644890).

### NC-6004 AND NC-4016

Besides paclitaxel, other drugs have also been incorporated into micelles for clinical purposes. NC-6004 (Nanoplatin^TM^) is a polymeric micelle comprised of PEG and poly(glutamic acid) with incorporated cisplatin, which is normally cleared rapidly via renal excretion after systemic administration, leading to nephrotoxicity (Gong et al., 2012). NC-6004 led to significant anti-tumor effects in a mouse model of colon adenocarcinoma 26, human gastric cancer (MKN-45) bearing mice, and in HT29 oxaliplatin-resistant bearing mice (Nishiyama et al., 2003; Uchino et al., 2005; Alami et al., 2006). In a phase I clinical trial, NC-6004 was used in the treatment of several solid tumors including lung, colon, hepatic, pancreatic, renal, melanoma, and esophageal cancers (Plummer et al., 2011). Out of 17 patients, 7 (41.2%) achieved stable disease. Renal impairment was still observed at high doses of the NC-6004, but in general, toxicities were less severe and less frequent when compared with cisplatin.

An additional micelle platform for the delivery of a platinum-based compound is NC-4016, a formulation consisting of PEG and a coordinate complex of poly amino acid and 1,2-diaminocyclohexane platinum (II) (Gong et al., 2012). A phase I clinical trial was started in March 2009 (Gong et al., 2012), but no updates have been published as of yet.

### SP1049C AND NK911

Doxorubicin has also been encapsulated into micelles for use in patients with solid tumors. SP1049C, a polymeric micelle consisting of poly(ethylene oxide)-poly(propylene oxide)-poly(ethylene oxide) block copolymer has been developed for this purpose (Gong et al., 2012). In a phase I clinical trial, SP1049C was used in the treatment of several solid tumors including colorectal, esophageal, lung, ovarian, kidney, and hepatic cancers in addition to soft-tissue sarcoma, mesothelioma, neuroblastoma, cholangiocarcinoma, and Ewing’s sarcoma (Danson et al., 2004). About 21 patients were evaluable for response, but no patients demonstrated complete or partial responses; 8 patients (31%) had stable disease. A phase II clinical study of SP1049C was conducted in patients with advanced adenocarcinoma of the esophagus and gastroesophageal junction (Valle et al., 2011). Out of 19 patients evaluable for response, 9 (47.4%) had partial responses and 8 (42.1%) had minor responses or stable disease. Median overall survival was 10 months, and neutropenia was found to be the principal toxicity of the compound. Another micelle formulation encapsulating doxorubicin is NK911, which consists of PEG-poly(Asp) block copolymers conjugated to Doxorubicin (Gong et al., 2012). In a phase I clinical study, NK911 was used to treat several other solid tumors including pancreatic, colorectal, esophageal, gall bladder, and stomach cancer as well as leiomyosarcoma (Matsumura et al., 2004). Out of 23 patients, 8 (34.7%) exhibited stable disease and 1 (4.3%) demonstrated a partial response. As with SP1049C, neutropenia was the primary hematologic toxicity.

### NC-6300

Another micelle system for clinical use may be just on the horizon. Takahashi et al. (2013) reported the use of NC-6300, an epirubicin-incorporated micelle, in mice bearing human hepatocellular carcinoma xenograft tumors. At doses of 10 and 15 mg/kg, NC-6300 led to significant survival improvements when compared to both control and epirubicin treated mice. The micellar formulation also appeared to decrease cardiotoxicity normally caused by epirubicin (Takahashi et al., 2013).

### NK102

**FIGURE 1:**
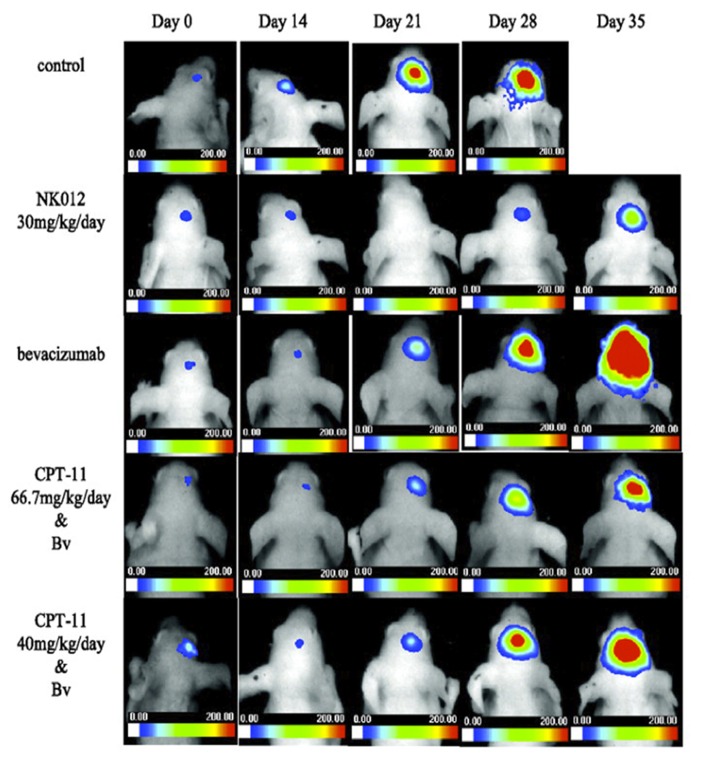
**Comparison of NK012 micelle formulation with bevacizumab and CPT-11 therapy.** Athymic mice were injected intracranially with Luciferase-labeled U87MG and treated starting 8 days after tumor cell implantation. NK012 was delivered at 30 mg/kg intravenously three times every 4 days. CPT-11 was delivered at 67 or 40 mg/kg three times every 4 days in conjunction with bevacizumab, which was delivered at 5 mg/kg intraperitoneally six times every 4 days. Reproduced with permission from Kuroda et al. (2010).

Unfortunately, these previously mentioned micelle formulations have not been applied to patients with GBM nor has there been any substantial preclinical testing in appropriate glioma animal models. However, NK012, a micelle composed of PEG-poly(Glu) block copolymer with covalently bound SN-38, has shown promise in the treatment of malignant gliomas. SN-38 is an active metabolite of CPT-11 (Irinotecan), a topoisomerase I inhibitor (Hsiang and Liu, 1988; Kawato et al., 1991; Gong et al., 2012). Kuroda et al. (2009) compared NK012 versus CPT-11 treatment in a U87MG xenograft mouse model. *In vitro* studies demonstrated that NK012 was 34 to 444-fold more potent than CPT-11 as tested in five different human glioma cell lines. *In vivo* studies demonstrated that NK012-treated (30 mg/kg/day) mice lived for a significantly longer time period when compared to both control (*p* = 0.001) and CPT-11-treated (66.7 mg/kg/day; *p* = 0.0014) mice (Kuroda et al., 2009). This group further expanded upon the study by examining the efficacy of NK012 ± bevacizumab (Kuroda et al., 2010). NK012 monotherapy (30 mg/kg/day) led to greater survival improvements in mice bearing U87MG orthotopic intracranial tumors when compared to any dosing method of CPT-11 in combination with bevacizumab (40 or 66.7 mg/kg/day CPT-11 + 5 mg/kg/day bevacizumab; *p* < 0.05). No difference was observed between NK012 mice and those treated with NK012 and bevacizumab (Kuroda et al., 2010). *In vivo* bioluminescence studies of these experiments are displayed in **Figure 1**. In terms of clinical trials, a phase I clinical study of NK012 for treatment of colorectal, pancreatic, and esophageal cancers as well as small cell, carcinoid, and non-small cell lung cancers was conducted (Hamaguchi et al., 2010). Out of 23 patients that were evaluable for response, 2 patients (8.7%) had partial responses and 5 patients (21.7%) maintained stable disease. In another phase I trial, Burris et al. (2008) reported that out of 16 evaluable patients treated with NK012, 2 (12.5%) were reported to have partial responses and 10 (62.5%) maintained stable disease. As with the other micelle formulations however, no micelle-drug combinations have been used in clinical studies to target GBM or other forms of brain cancer.

## MAJOR OBSTACLES TO THE IMPLEMENTATION OF MICELLE-BASED GBM THERAPY

It is alarming that while these micelle formulations have been successfully applied to many different types of solid tumors in both preclinical and clinical settings, their use for the treatment of GBM is still absent. There are, however, some significant hurdles that may be preventing their application in the context of GBM therapy:

(1) As these cells are located within the CNS, systemically administered therapeutic agents must cross the BBB to reach target cancerous tissue. Despite the presence of a compromised vasculature that may increase the intratumoral EPR effect (Fang et al., 2011), many therapeutic agents still do not reach significantly toxic levels within tumors.
(2) GBM tumors are heterogeneous entities with some areas of necrotic and hypoxic tissue and other areas containing neovascularization. Necrotic pockets, areas of fibrosis, and hypovascularization are major causes of decreased intratumoral distribution of therapeutic agents while hypervascularized areas encourage accumulation in surrounding tissue. An understanding of nanoparticle distribution within a tumor is important as certain populations of cells, such as GBM cancer stem cells that self-renew and maintain a tumor, may have specific vascular niches in which they are located (Calabrese et al., 2007; Gilbertson and Rich, 2007).
(3) There are inherent weaknesses to therapeutic delivery depending on the route of administration. Many systemically administered therapeutic agents suffer from rapid clearance from circulation by the reticuloendothelial and cause non-specific toxicity to organs. Therefore, improvements in drug-circulation time and specificity of targeting are important steps forward for this route of administration. Intratumoral administration of therapeutic agents is limited due to high interstitial pressures that cause poor dissemination of molecules (Jain, 1989) and difficulties in delivering multiple dosing regimens to a patient.
(4) Although these clinical micelle formulations enhance drug potency in many different solid tumor types, they currently do not possess any targeting moieties that could allow for greater CNS or brain tumor specific accumulation. Molecular targeting of different receptors expressed on glioma cells may be needed to improve the efficacy of currently available formulations.
(5) If large quantities of administered micelles are needed to ensure adequate intratumoral accumulation, systemic toxicity may inevitably be an issue due to the lack of a controlled-release function. Thus, it would be appropriate to incorporate stimulus-triggered releasing mechanisms of entrapped agents, which would enable release only within the tumor vicinity, while minimizing non-specific release before arrival to the tumor site.

## STIMULUS-TRIGGERED RELEASE OF THERAPEUTIC AGENTS

Controlled release in the field of nanomedicine can allow for more specific delivery of therapeutic cargos to a tumor site. This may be especially important for systemic delivery in the context of brain tumor treatment, where there is a desire to prevent premature drug release prior to delivery across the BBB. Here, we differentiate between release mechanisms that rely on processes that are intrinsic to tumor cells or the tumor microenvironment (“internal” triggers) and release mechanisms that can be initiated by processes external to the body (“external” triggers). A summary of these release mechanisms is presented in **Table 1**.

**Table 1:** Stimulus-triggered release incorporated mechanism for micelle particles.

Type	Stimulus	Linker/release mechanism	Examples used in micelles
Internal	pH	Ortho ester	Tang etal. (2011)
		Hydrazone bond	Bae etal. (2003), Lee etal. (2012), Xiao etal. (2012)
		Cis-aconityl bond	Yoo etal. (2002)
		Acetal bond	Gillies and Frechet (2003)
	Reduction	Disulfide bond	Heffernan and Murthy (2009), Xu etal. (2009), Abdullah Al etal. (2011)
	Enzyme-mediated	Cleavage by MMP-2/9	Gu etal. (2013)
External	Ultrasonography	Micelle structure perturbation	Rapoport (2004)
		Release of micelles after rupture of gas liposome	Yin etal. (2013)
	Temperature	Disruption of interactions between thermosensitive copolymers	Soga etal. (2005), Bae etal. (2006), Yang etal. (2007), Prabaharan etal. (2009), Talelli etal. (2011), Shi etal. (2013)
	Light	Transformation of hydrophobic DNQ to hydrophilic 3-indenecarboxylic acid	Goodwin etal. (2005), Sun etal. (2012)

*MMP-2/9, Matrix metalloproteinase 2/9; DNQ, 2-Diazo-1,2-naphthoquinone.*

### INTERNAL TRIGGERS

The major releasing mechanisms that fall under this category are pH-sensitive and reduction-responsive release. It is known that the tumor microenvironment is slightly acidic with a pH of about 6.5–7.2 (Webb et al., 2011; Du et al., 2013). In addition to the microenvironment, acidic compartments within cells such as endosomes and lysosomes have a pH around 4.5–5.5 (Fehrenbacher and Jäättelä, 2005; Du et al., 2013). The low pH in such compartments can be a powerful tool for enabling extensive drug release, especially for particles that are taken up via the endocytic pathway. For example, micelles composed of pH-sensitive copolymer such as PEG-poly(L-histidine) or pH-labile hydrazone linkers are stable during the circulation in the blood; yet, after accumulation in the tumor, they can be dissembled into copolymers and release encapsulated drugs (Gaucher et al., 2005). Different pH-sensitive conjugation linkers have been developed for micelle systems including acid labile ortho esters (Tang et al., 2011), hydrazone bonds (Bae et al., 2003; Lee et al., 2012; Xiao et al., 2012), cis-aconityl bonds (Yoo et al., 2002), and acetal bonds (Gillies and Frechet, 2003). Yoo et al. (2002) compared doxorubicin release from micelles after using either a hydrazone or cis-aconityl bond for drug conjugation. For micelles possessing cis-aconityl bonds, less than 10% release was observed at pH 7 by 24 h whereas at a pH of 5, roughly 50% of the drug was released. For micelles possessing a hydrazone linkage for drug incorporation, about 30% drug release was observed by 16 days at pH 7 whereas close to 100% of doxorubicin was released at a pH of 5 (Yoo et al., 2002).

Another intrinsic release mechanism that has been investigated involves reduction-mediated release of therapeutic agents from disulfide-cross-linked micelles. Micelles possessing this crosslinking are more stable in circulation but upon internalized into a cell and exposure to high levels of glutathione in this environment, the stability of the system is disrupted, facilitating drug release (Heffernan and Murthy, 2009; Xu et al., 2009). Abdullah Al et al. (2011) described thiolated pluronic micelles with cores formed by disulfide bonds of functionalized Pluronic F127, a PEO-PPO-PEO triblock copolymer. At increasing concentrations of the reducing agent dithiothreitol (DTT), increasing paclitaxel release was observed. Heffernan and Murthy (2009) developed micelles composed of PEG-PLL block copolymer that were modified with cross-linkable dithiopyridine groups. These micelles were used to deliver proteins such as antigen ovalbumin and catalase as well as CpG-DNA. The proteins mentioned were modified with dithiopyridine moieties as well to allow for tethering to the core of micelles (Heffernan and Murthy, 2009). Xu et al. (2009) reported the synthesis of reduction-sensitive cross-linked micelles for triggered release of doxorubicin. When no DTT was present, only ~10% doxorubicin release was observed in cross-linked micelles at 10 h. However, at 10 mM DTT exposure, ~75% doxorubicin release was observed by 9 h (Xu et al., 2009).

**FIGURE 2:**
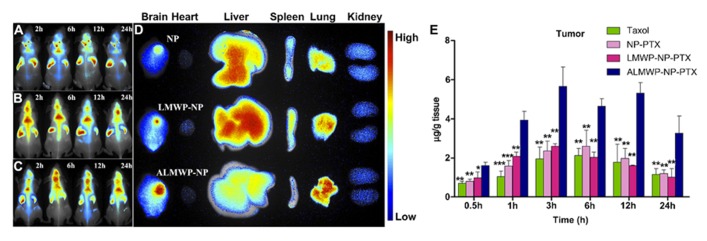
**Activatable low molecular weight protamine (ALMWP)-modified micelles allow for increased accumulation of paclitaxel in an intracranial C6 glioma model in nude mice. (A–C)** *In vivo* fluorescence imaging taken of nude mice bearing intracranial C6 glioma tumors. Mice were injected intravenoulsy with DiR-labeled micelles **(A)**, LMWP-micelles **(B)**, and ALMWP-micelles **(C)**. **(D)** Images of organs taken from mice sacrificed 24 h after intravenous injection of the various micelle particles. **(E)** Accumulation of intravenously injected compounds at the tumor site from 0.5 to 24 h after administration. *p < 0.05, **p < 0.01, ***p < 0.001 was indicative of significant difference between the experimental and ALMWP-NP-PTX group. Error bars reflect the standard deviation. Reproduced with permission from Gu et al. (2013).

Enzyme-mediated activation is also an attractive mechanism for therapeutic activation of micelles. Gu et al. (2013) developed a different strategy for glioma targeting by designing micelles modified with a cell penetrating peptide (low molecular weight protamine, LMWP) that could be specifically activated when MMP-2 and 9 were present in the environment. MMP-2 and MMP-9 are overexpressed by glioma cells as well as the tumor vasculature (Forsyth et al., 1999). The positive charges on the LMWP were masked by a polyanionic peptide with an MMP-2/9-cleavable peptide linker sequence (PLGLAG). This activatable LWMP (ALMWP) was conjugated to micelles, allowing for delivery of paclitaxel. In mice bearing intracranial C6 glioma tumors, ALWMP micelles carrying paclitaxel led to significantly longer survival times when compared to Taxol (*p* < 0.01) as well as LMWP-modified micelles (*p* < 0.05). *In vivo* fluorescence imaging after micelle administration and quantification of intratumor accumulation of paclitaxel is displayed in **Figure 2**.

Dual-responsive micelles that can release doxorubicin upon exposure to low pH and a reductive environment have already been investigated (Chen et al., 2013) and may allow for even more release specificity and increased stability of particles in circulation. These micelles, composed of PEG-SS-poly(2,4,6-trimethoxybenzylidene-pentaerythritol carbonate) block copolymer, only allowed for ~24.5% doxorubicin release by 21 h at pH 7.4. However, upon exposure to both 10 mM GSH and pH 5.0, 94.2% doxorubicin release could be achieved.

### EXTERNAL TRIGGERS

External triggers such as ultrasonography (US), heat, and light are alternative approaches for mediating micellar drug release (Marin et al., 2002; Rapoport, 2004). An important advantage of external triggers is that the drug release may be controlled locally and in a time-dependent manner, which can improve drug uptake into target tissues while minimizing systemic toxicity. One method used to externally mediate release is US, a non-invasive imaging technique. Yin et al. (2013) designed ultrasound-sensitive nanobubbles for the delivery of siRNA targeting sirtuin 2 (SIRT2), an anti-apoptotic gene. Nanobubbles were composed of a hetero-assembly of siRNA-loaded polymeric micelles and liposomes. Positively charged micelles were first loaded with siRNA, which were then loaded onto negatively charged gas-cored liposomes. Upon exposure to low-frequency US, siRNA-loaded micelles could be released from their electrostatic interaction with liposomes. *In vivo* experiments demonstrated that mice with subcutaneous C6 glioma tumors displayed both smaller tumor volume and improved survival when treated with this system in combination with low frequency US (Yin et al., 2013).

Local heating of a tumor can be achieved by various methods including continuous wave ultrasound (Rapoport, 2007) as well as other hyperthermia-inducing instruments. Thus, thermosenstive crosslinking may also increase the efficiency of drug delivery to a particular site. Several studies have already demonstrated thermosensitive micelles incorporating therapeutic agents (Soga et al., 2005; Bae et al., 2006; Yang et al., 2007; Prabaharan et al., 2009; Talelli et al., 2011; Shi et al., 2013). For example, Yang et al. (2007) described micelles composed of a novel thermosensitive poly(*N*-isopropylacrylamide-co-acrylamide)-*b*-poly(D,L-lactide) copolymer which was stable up to 41°C. Docetaxel release at 43°C was ~90% by 70 h versus ~50% release at 37°C (Yang et al., 2007). Micelles have even been combined with other particle types to achieve thermosensitive volume changes that could allow for transient release of incorporated agents (Bae et al., 2006).

Light-mediated release may also be a valuable mechanism for drug unloading. Goodwin et al. (2005) demonstrated that micelles with incorporated 2-diazo-1,2-naphthoquinones (DNQ) could undergo dissociation in response to ultraviolet and infrared light. Recently, diazonaphthoquinone-cored amphiphiles assembled from Janus-type poly(amido amine) dendrimers responding to near-infrared light were described (Sun et al., 2012). Release of doxorubicin nearly doubled after 10 min of exposure to 808 nm laser irradiation compared to non-irradiated particles.

## GLIOMA-SPECIFIC TARGETING MOIETIES

As mentioned previously, a major (perhaps the greatest) limitation of systemically administered unmodified micelles is their impaired penetration through the BBB. Although the EPR effect can allow for some accumulation of particles at a brain tumor site, drug concentrations delivered may still be sub-therapeutic. A solution to this issue is to covalently attach targeting molecules onto the surface of drug-loaded micelles to improve localization to a tumor. A summary of the following targeting-moieties as well as others not discussed is presented in **Table 2**.

**Table 2:** Targets and targeting moieties to enhance micelle specificity toward brain tumors.

Target	Target location	Targeting molecule	Examples of incorporation onto micelles
α_v_β_3_ integrin	Tumor vasculature (Bello etal., 2001) Glioma cells (Bello etal., 2001)	RGD peptide (Arap etal., 1998)	Nasongkla etal. (2006), Oba etal. (2007), Hu etal. (2008), Zhan etal. (2010), Kessinger etal. (2011), Liu etal. (2012), Xiao etal. (2012), Zhan etal. (2012a,b), Zhang etal. (2012a),
Fibrin deposits	Tumor vasculature (Simberg etal., 2007)	CREKA Peptide (Simberg etal., 2007)	Chung etal. (2013), Peters etal. (2009)
	Tumor stroma (Bardos etal., 1996; Simberg etal., 2007)		
Aminopeptidase N	Tumor vasculature (Pasqualini etal., 2000)	NGR peptide (Ellerby etal., 1999; Pasqualini etal., 2000)	Zhao etal. (2011)
	BBB pericytes (Kunz etal., 1994)		
Transferrin receptor	CNS vasculature (Fishman etal., 1987)	Transferrin (Fishman etal., 1987)	Ren etal. (2010), Zhang etal. (2012a,b)
		Lactoferrin (Liu etal., 2013)	Liu etal. (2013)
		Aptamer (Mu etal., 2013)	Mu etal. (2013)
nAchR	CNS vasculature (Kalaria etal., 1994; MacKlin etal., 1998)	Candoxin-derived peptide (Zhan etal., 2011)	Zhan etal. (2011,2012b)
EGFR	Glioma cells (Wong etal., 1987)	Anti-EGFR Antibody (Kuo and Liang, 2011)	Kuo and Liang (2011)
		EGa1 (Oliveira etal., 2010)	Talelli etal. (2011)
LRP1	Glioma cells (Bu etal., 1994; Yamamoto etal., 1997)	Angiopep-2 (Demeule etal., 2008)	Shen etal. (2011)
	Neurons (Herz and Bock, 2002)		
Unknown	Glioma cells (Bayrac etal., 2011)	GMT8 aptamer (Bayrac etal., 2011; Gao etal., 2012)	Gao etal. (2012)

*RGD, Arg-Gly-Asp; NGR, Asn-Gly-Arg; BBB, Blood brain barrier; CNS, Central nervous system; EGFR, Epidermal growth factor receptor; LRP1, Low-density lipoprotein receptor-related protein 1.*

Incorporation of the Arg-Gly-Asp (RGD) tri-peptide is such an example of a glioma targeting strategy. The RGD peptide binds to α_v_β_3_ integrin, a receptor that is overexpressed on both tumor cells as well as on the tumor vasculature (Bello et al., 2001), with high affinity (Arap et al., 1998). Several studies have incorporated the cyclic RGD peptide (cRGD) into their micellar systems to target GBM. Zhan et al. (2012a) designed a cRGD-PEG-PEI polymeric micelle for delivery of the gene for tumor necrosis factor-related apoptosis-inducing ligand (pORF-hTRAIL). Using this targeting moiety, targeted gene delivery in an intracranial U87 mouse model could be achieved with a higher gene transfer efficiency compared to unmodified particles. This targeted system led to prolonged survival in these mice (23.5 vs. 19 days; *p* < 0.05) along with higher TRAIL expression levels (Zhan et al., 2012a). This same group later delivered cRGD-PEG-PEI/pORG-hTRAIL particles in conjunction with candoxin-derived peptide-modified PEG-PLA micelles loaded with paclitaxel to mice bearing intracranial GBM tumors (Zhan et al., 2012b). A candoxin derivative was chosen as it has previously been shown to target nicotinic acetylcholine receptors expressed on the BBB (Zhan et al., 2011). Paclitaxel was found to increase the transfection of the TRAIL gene into U87 cells, thus increasing the apoptotic effect when combining the two agents (Zhan et al., 2012b). Jiang et al. (2013) specifically studied the penetrating depth of cRGD-modified poly(trimethylene carbonate)-based micelles carrying paclitaxel into glioma tissue as well as systemic particle distribution after intravenous administration in an intracranial U87MG mouse model. cRGD modification was found to enhance micellar penetration into U87MG glioma spheroids in culture as well as into intracranial tumors *in vivo*. Furthermore, these particles led to an increase in the median survival of U87MG glioma-bearing mice (32 days) when compared to both non-modified micelles carrying paclitaxel (27 days, *p* = 0.012) as well as Taxol (23 days, *p* < 0.001) (Jiang et al., 2013). Xiao et al. (2012) developed cRGD modified micelles conjugated to doxorubicin (via a pH-sensitive hydrazone bond) and 1,4,7-triazacyclononane-*N,N*′, *N*″-triacetic acid (NOTA), a macrocyclic chelator for ^64^Cu labeling and PET imaging. Such a system allowed for the quantitative measurement of *in vivo* particle distribution in a front flank U87MG mouse model. About 5.7% ID/g was observed in the tumor 4 h after injection of cyclic-RGD modified micelles, which was significantly higher than non-modified micelles. Besides the tumor site, particle deposition was seen highest in the liver, kidney, lungs, and intestines (Xiao et al., 2012). Kessinger et al. (2011) also examined the targeting kinetics of cRGD-modified micelles loaded with ultra-sensitive SPIO nanoparticles. α_v_β_3_-specific accumulation of these particles was observed in subcutaneous U87 tumors within the first 5 min after administration, with an accumulation rate of 0.24 min^-^^1^ when using a one-compartment pharmacokinetic model (Kessinger et al., 2011).

Another promising target for nanomedicines are fibrin deposits within the tumor vasculature and stroma. Such deposits are found to be distributed throughout primary and metastatic brain tumors (Bardos et al., 1996) and have recently been a target of cysteine-arginine-glutamic acid-lysine-alanine (CREKA) peptide-modified micelles. Chung et al. (2013) demonstrated that Cy7-labeled CREKA-micelles could accumulate to a greater extent in GL261 glioma bearing mice at 3 h and 24 h after administration when compared to micelles without the CREKA modification. Future work with CREKA-micelles could aim to deliver therapeutic agents in GBM animal models to test for survival improvements.

Yet another targeting strategy for micelles is the incorporation of transferrin (Tf) into their structure (Ren et al., 2010; Zhang et al., 2012a,b). Tf is normally transported into the CNS via a Tf receptor-mediated pathway in endothelial cells (Fishman et al., 1987). Zhang et al. (2012b) synthesized Tf-modified polyphosphoester hybrid micelles containing paclitaxel for use in the treatment of mice bearing intracranial U87MG tumors. Mice treated with Tf-modified micelles showed significantly prolonged survival (39.5 days) when compared to animals treated with Taxol (33.6 days, *p* < 0.01). Biodistribution studies showed greater %ID/g reaching brain tissue with transferrin functionalization. However, this amount (~2.5 × 10^-^^4^ %ID/g) was very small in comparison with other body organs including the liver (~15 %ID/g), spleen (~5 %ID/g), lungs (~4 %ID/g), and kidney (~5 %ID/g). Thus, even with functionalization, penetration into the CNS was still poor. The same group also developed Tf-modified micelles loaded with cRGD-paclitaxel conjugates in an effort to take advantage of both a BBB crossing pathway and a tumor-specific targeting mechanism (Zhang et al., 2012a). This system led to a significant improvement in mean survival time in mice bearing intracranial U87MG tumors (42.8 days) when compared to Tf-modified paclitaxel-loaded micelles (39.5 days, *p* < 0.05), paclitaxel-loaded micelles (34.8 days), and Taxol (33.6 days). Again, biodistribution studies showed improved intratumoral accumulation of particles when both targeting components were used (~0.7 %ID/g at 4 h), but these levels still paled in comparison to the amount reaching the liver (~10 %ID/g), spleen (~4 %ID/g), and lungs (~4 %ID/g).

Besides peptide-based moieties, aptamers have also been used to aid in the targeting of glioma cells. Gao et al. (2012) functionalized micelles with GMT8 aptamers that were selected by a cell-based systematic evolution of ligands by exponential enrichment (SELEX) method (Shangguan et al., 2006) and were shown to specifically bind to U87 cells. Aptamer-modified micelles were able to penetrate U87 tumor spheroids more effectively and, when loaded with docetaxel, led to improved mean survival time (40 days), which was significantly longer than unmodified micelles carrying docetaxel (35 days, *p* < 0.05) and free docetaxel (30 days, *p* < 0.05; Gao et al., 2012).

## DELIVERY OF THERAPEUTIC MICELLES TO BRAIN TUMORS

**FIGURE 3:**
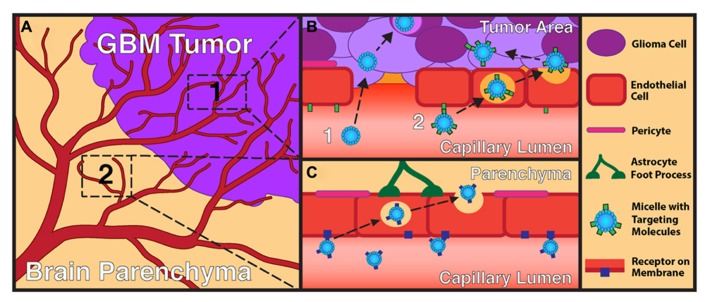
**Overview of GBM-targeting mechanisms for micelle nanoparticles. (A)** Section of brain containing a GBM tumor and normal brain parenchyma. **(B)** Close-up of tumor vasculature and surrounding glioma cells. Here, the vasculature displays disruption of the tight junctions between endothelial cells. Micelles can target tumors areas by two main pathways: (1) via the EPR effect where micelles diffuse passively through the disrupted BBB to reach glioma cells or (2) via interaction with endothelial cells and transcytosis to the tumor parenchyma. Examples of receptors more specific to the tumor vasculature and glioma cells include α_v_β_3_ integrin and aminopeptidase N. **(C)** Close-up of normal vasculature with surrounding normal brain parenchyma. Here, micelles can interact with the intact BBB, allowing for transcytosis of particles. An example of a receptor mediating this pathway includes the Tf receptor. While receptor-mediated endocytosis is displayed in these images, other endothelial cell uptake mechanisms such as adsorptive-mediated endocytosis may take place at these sites as well.

Micelles can be conveyed to a brain tumor site either by systemic or local administration. Both methods present their own advantages and drawbacks. Systemically administered drug-loaded micelles represent a very attractive delivery method due to their relatively non-invasive nature. **Figure 3** illustrates how particles can reach the CNS and a brain tumor after systemic delivery due to both the EPR effect as well as transportation by endothelial cells into the parenchyma. However, surface modification is essential for this administration method as unmodified micelles may be rapidly cleared from the bloodstream upon intravenous administration due to antibody opsonization. These opsonized particles are engulfed by macrophages from the reticuloendothelial system and remain trapped in the liver or spleen, decreasing their therapeutic efficacy (Pardridge, 1992). To avoid such uptake, micelles have been engineered with a reduced size (typically < 100 nm), hydrophilic blocks such as PEG (Calvo et al., 2001; Brigger et al., 2002), or additional coating surfactant (Gelperina et al., 2010). This hydrophilic surface significantly decreases complement activation and macrophage recognition. Consequently, there is a considerable increase in their vascular circulation time, with an enhanced delivery of these particles to the tumor burden (Tiwari and Amiji, 2006).

Although modified micelles have proven to effectively cross the BBB, accumulate within a tumor site, and increase animal survival after intravenous administration, they still present important shortcomings such as uneven intratumoral distribution due to the variability in vasculature within a GBM tumor and widespread deposition of particles in non-cancerous areas such as the liver, spleen, and lungs. Other avenues of delivery aim to overcome these limitations. Kanazawa et al. (2011) demonstrated the delivery of coumarin 6 to the brain of C6 glioma bearing rats by micelles modified with Tat, a cell penetration peptide, after intranasal administration. About 1 h after intranasal administration, ~1.6 %ID/g was observed in either hemisphere of the brain (Kanazawa et al., 2011). It is important to note that intranasal delivery of micelles did not lead to preferential accumulation within the tumor side of the brain, suggesting that the EPR effect was not connected with this delivery method. Liu et al. (2013) developed a “nose-to-brain” micellar delivery system for neuroprotective peptides targeted against Alzheimer’s diseases. Lactoferrin was conjugated onto PEG-PCL micelles. This group reported the localization of coumarin-6 loaded lactoferrin-modified micelles to the olfactory bulb, olfactory tract, hippocampus, cerebellum, and cerebrum after intranasal administration. This method led to improved memory performance in a Morris water maze experiment as well as diminished acetylcholinesterase and improved choline acetyltransferase activity. Although this method was not used in a brain tumor model, it suggests that a similar set-up involving delivery of glioma toxic agents may be a viable avenue for exploration.

In contrast to intravenous or intranasal delivery, local administration of drug-loaded micelles can achieve much higher concentrations of nanoparticles at the tumor site. The commonly observed problems of injection backflow and limited intratumoral diffusion using this method can be overcome by CED, a technique that uses a pressure gradient to supplement local diffusion so as to achieve an efficient intratumoral distribution of the injected compound (Bobo et al., 1994; Allard et al., 2009). As a result, CED is able to treat larger areas of brain as opposed to local diffusion alone (Lesniak, 2005). Albeit invasive, it has been successfully used in multiple clinical trials as a therapeutic approach for glioma patients (Sampson et al., 2003; Weaver and Laske, 2003). Preclinical data using CED for paclitaxel-loaded nanoparticle delivery has displayed enhanced animal survival in glioma xenograft models (Vinchon-Petit et al., 2010). However, this approach presents some important disadvantages. First, infusion of big volumes can invariably cause increased intracranial pressure. Second, although CED leads to a more even intratumoral spread, drug distribution is still unpredictable. Lastly, prolonged intracranial infusion may induce local infection, since it increases the exposure of brain tissues to the external environment.

An alternative to using CED is loading these therapeutic agents into stem cell carriers, such as mesenchymal stem cells or neural stem cells. These vehicles possess intrinsic immunosuppressive and tumor-tropic properties that can lead to intratumoral distribution and targeted delivery to infiltrative tumor areas without toxicity to non-neoplastic tissues (Thaci et al., 2012; Huang et al., 2013). Although stem cells have not been used yet for the delivery of micelles specifically, they have been employed as carriers for other nanoparticle-drug conjugate systems (Roger et al., 2010; Li et al., 2011; Cheng et al., 2013).

## CONCLUSION

Polymeric micelles offer great potential in the area of therapeutic delivery as has already been demonstrated in the context of several solid tumor diseases. However, the incorporation of glioma-specific targeting moieties and controlled drug release mechanisms needs to occur if these particles are going to be effective at targeting GBM tumors. Alternatively, local administration methods could be utilized, but such a method for delivery of loaded-micelles would need to demonstrate improved efficacy over infusing a therapeutic agent alone. With the proper allocation of time and resources to developing micellar-based therapeutics for GBM, we may see clinical applications of these systems for brain tumors in the not too distant future.

## AUTHOR CONTRIBUTIONS

Ramin A. Morshed, Yu Cheng, Brenda Auffinger, Michelle L. Wegscheid, and Maciej S. Lesniak all contributed to the manuscript and participated in the editing process. In addition, Maciej S. Lesniak oversaw all aspects of writing and revision.

## Conflict of Interest Statement

The authors declare that the research was conducted in the absence of any commercial or financial relationships that could be construed as a potential conflict of interest.
